# Therapeutic Resistance in Acute Myeloid Leukemia: The Role of Non-Coding RNAs

**DOI:** 10.3390/ijms17122080

**Published:** 2016-12-10

**Authors:** Armin Zebisch, Stefan Hatzl, Martin Pichler, Albert Wölfler, Heinz Sill

**Affiliations:** 1 Division of Hematology, Medical University of Graz, 8036 Graz, Austria; stefan.hatzl@medunigraz.at (S.H.); albert.woelfler@medunigraz.at (A.W.); heinz.sill@medunigraz.at (H.S.); 2 Division of Oncology, Medical University of Graz, 8036 Graz, Austria; martin.pichler@medunigraz.at

**Keywords:** acute myeloid leukemia, risk stratification, chemoresistance, micro-RNA, long non-coding RNA

## Abstract

Acute myeloid leukemia (AML) is caused by malignant transformation of hematopoietic stem or progenitor cells and displays the most frequent acute leukemia in adults. Although some patients can be cured with high dose chemotherapy and allogeneic hematopoietic stem cell transplantation, the majority still succumbs to chemoresistant disease. Micro-RNAs (miRNAs) and long non-coding RNAs (lncRNAs) are non-coding RNA fragments and act as key players in the regulation of both physiologic and pathologic gene expression profiles. Aberrant expression of various non-coding RNAs proved to be of seminal importance in the pathogenesis of AML, as well in the development of resistance to chemotherapy. In this review, we discuss the role of miRNAs and lncRNAs with respect to sensitivity and resistance to treatment regimens currently used in AML and provide an outlook on potential therapeutic targets emerging thereof.

## 1. Introduction

Acute myeloid leukemia (AML) is an aggressive malignancy of the hematopoietic system, which is caused by malignant transformation of hematopoietic stem- or progenitor cells [1,2,3]. AML may occur “de novo”, secondary to antecedent hematological disorders or after chemo- and/or radiotherapy for a primary disease [1,2,4,5]. AML is the most common form of acute leukemia in adults with an incidence of approximately 2–4/100,000 per year (http://seer.cancer.gov/) [1,6]. Although everyone can be affected by this disorder, it is mainly a disease of the elderly with a median age at diagnosis of 72 years (http://seer.cancer.gov/). With current high-intensity treatment approaches, approximately 35%–40% of younger AML patients can be cured, however, in older patients >60 years of age, long lasting remissions can be achieved in up to 15% of cases only [1,2,7]. Unfortunately, outcomes are even worse in very old patients, or in those with additional comorbidities excluding them from high-dose approaches. Although the introduction of low-intensity therapeutic regimens (LITR), in particular the hypomethylating agents (HMA) azacitidine and decitabine, has resulted in both increased response rates and extended survival within this cohort, the vast majority of these patients ultimately succumbs to therapy-resistant, progressive disease [1,8,9]. Prognosis of AML patients is influenced by patient-associated factors on the one hand and by disease-related factors on the other hand. Particularly, research on disease-related factors has made significant progress in the last years, as a variety of molecular markers with prognostic relevance have been identified via the introduction of next generation sequencing techniques. This has led to the development of robust risk stratification models that have helped to accurately predict the clinical course of a given patient and to plan the therapeutic approach accordingly [1,7,10]. Unfortunately, despite all this progress in diagnosis and risk evaluation, the major problem in AML is still the development of chemorefractory disease. Although a large proportion of patients achieves complete remission (CR) after cytotoxic therapy, a significant subset presents with primary refractory disease. Even in patients achieving CR, a substantial subgroup ultimately develops chemoresistant relapse [1,6,7,11,12], which is caused by the fact that cytotoxic therapy failed to eradicate a therapy-resistant (sub)clone within these patients [13]. In any case, the vast majority of these patients still succumbs to this complication, making chemoresistance one of the primary targets of AML research with a tremendous potential to increase survival if overcome.

Non-coding RNAs (ncRNAs) are RNA transcripts of variable length, which are basically not transcribed and translated into proteins. Quite frequently, they are produced from introns, intergenic stretches or from antisense transcripts of protein coding genes [14,15,16,17]. Given the fact that only a minority of RNA transcripts within mammalian cells is truly transcribed into protein-coding mRNAs, and that the function of ncRNAs has been unknown for a long time, they have been considered as genomic debris initially. However, it is now evident, that they play a central role in many cellular functions, primarily by regulation of gene expression levels. This is orchestrated in a variety of ways, including inhibition of transcription and translation, as well as by epigenetic modification and destabilization of protein coding genes [14]. Although a broad range of ncRNAs has been discovered, the probably best studied examples, especially in cancer, are micro-RNAs (miRNAs) and long non-coding RNAs (lncRNAs). While miRNAs are usually only 19–24 nucleotides long, lncRNAs display a length of more than 200 nucleotides. Aberrant expression of miRNAs and lncRNAs has been described in all types of cancers, and their functional involvement in pathogenesis of these malignant disorders is continuously being unraveled [15,16]. In AML, aberrant expression of ncRNAs has also been described and shown to be a pivotal process in malignant transformation of hematopoietic stem and/or progenitor cells [18]. Aberrant ncRNA expression thereby often acts as part of complex networks which additionally includes other genetic alterations in oncogenes and/or tumor-suppressors, as well as altered activation of transcription factors and signaling cascades. Quite frequently, ncRNAs thereby influence the activation status of these networks by targeting one or more of their members in a complex manner. On the other hand, expression of ncRNAs is frequently under the control of other players within these networks as well, thereby forming complex circuits which finally orchestrate malignant transformation. Among others, these networks include prominent players in leukemogenesis, such as *fms-like tyrosine kinase 3* (*FLT3*), *nucleophosmin* (*NPM1*), *CCAAT/Enhancer Binding Protein Alpha* (*CEBPA*), *tumor protein P53* (*TP53*) or the RAS-mitogen-activated protein kinase/extracellular signal-regulated kinase (RAS-MAPK/ERK) signaling cascade [19,20,21,22,23]. Lately, the role of ncRNA in mediating sensitivity and resistance to cytotoxic agents, respectively, has been discovered, which makes them attractive targets of novel anti-cancer therapies. Here, we review the role of miRNAs and lncRNAs in therapeutic resistance of AML and discuss potential therapeutic options arising thereof.

## 2. miRNAs and lncRNAs in Sensitivity and Resistance to High-Intensity Therapeutic Regimens

In patients eligible to high-intensity therapeutic regimens, induction chemotherapy consisting of continuous infusion cytarabine over seven days, usually combined with three days of an anthracycline (in most cases daunorubicin) is administered. This so-called “3 + 7” scheme has been largely unchanged for 30 years now and results in CR rates in 60% to 85% in patients younger than 60 years and 40% to 60% in patients older than 60 years [1,7]. In the case CR is achieved, consolidation therapy has to be employed. Currently, postremission consolidation comprises two to four cycles of intermediate-dose cytarabine or allogeneic hematopoietic stem cell transplantation (HSCT) [1,7]. Whether a patient receives cytarabine consolidation or HSCT mainly depends on the risk profile of the respective AML. In this respect, a risk stratification model has been proposed by the European LeukemiaNet and has evolved as widely accepted standard since [7]. This model is based on cytogenetic and molecular information, subdividing AML patients into four groups (favorable, intermediate-I, intermediate-II and adverse). While cytarabine consolidation is the treatment of choice for patients within the favorable group who achieve CR after induction therapy, allogeneic HSCT has to be considered for all other cases [1,7,10,24]. In the case chemoresistant disease or relapse after CR occurs, a variety of re-induction schemes have been tested. In the case the patient is still eligible for high-intensity approaches, they all aim to induce AML remission with subsequent allogeneic first (or second) HSCT [1]. Unfortunately, however, outcomes within these patients are dismal, which highlights the need for novel therapeutic approaches with the potential to overcome therapeutic resistance.

### 2.1. The Role of miRNAs

A wide range of studies dealing with miRNA expression and therapeutic resistance to intensive AML treatment have been published and allow classification of miRNAs into two major groups (Table 1). Group I comprises miRNAs, in which increased expression mediates sensitivity to chemotherapy, or, on the contrary, in which decreased expression mediates resistance. Group II comprises miRNAs, in which increased expression relays therapeutic resistance, whereas decreased expression mediates sensitivity.

**Figure 1:**
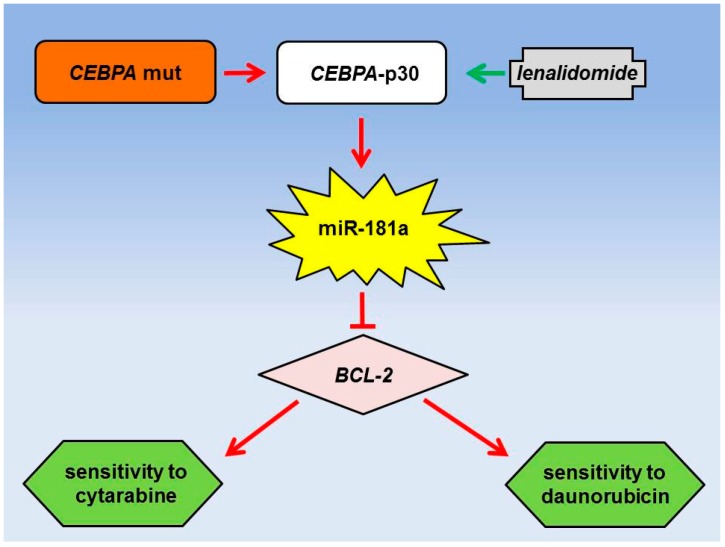
Example of a miRNA network influencing therapeutic sensitivity in acute myeloid leukemia (AML) high-intensity treatment. N-terminal mutations in *CEBPA* enable translation of a truncated *CEBPA*-p30 isoform, which in turn induces miR-181a [58]. As a consequence, *BCL-2*—a direct miR-181a target—is downregulated, which in turn increases the sensitivity to both cytarabine and daunorubicin [49,50,58]. Interestingly, expression of *CEBPA*-p30 is induced by lenalidomide as well [58].

One of the first insights into group I were revealed via a landmark paper by Marcucci and coworkers, who studied diagnostic leukemia specimens from AML patients treated within Cancer and Leukemia Group B (CALGB) trials [25]. Their analyses comprised a training group of 64 and a validation group of 55 adult patients <60 years with a normal karyotype and high-risk molecular features. By employing expression arrays with 305 miRNA probes, they were able to identify that increased expression of the miR-181 family was associated with a favorable clinical outcome (Figure 1). These results were later corroborated in another CALGB analysis, studying the expression of miR-181a in 309 diagnostic cytogenetically normal (CN) AML specimens [26]. Again, miR-181a proved to be a favorable prognostic marker and in addition to the previous study, the authors were also able to demonstrate, that the favorable prognostic value of miR-181a did not only apply to younger patients with molecular high-risk features, but also to an older cohort comprising patients >60 years of age. Most importantly, however, as all patients were treated on similar high-dose induction protocols and as high miR-181a expression was associated with achievement of CR in uni- and multivariate analyses, these data suggested a potential impact of this miRNA on mechanisms of sensitivity to AML high-intensity chemotherapeutic regimens. In this respect, the authors tried to identify potential target genes by performing mRNA arrays and correlating the results to the expression of miR-181a. In total, 1174 probe sets showed a statistically significant correlation, which was inversely in 1002 of them, thereby making them potential targets of miR-181a. Although functional experiments were missing, this list included interesting candidates, such as the *homeobox* (*HOX*) *A* and *B* clusters, whose overexpression had been linked to myeloid leukemogenesis and adverse prognosis in AML previously [57]. This correlation, as well as its prognostic value could later be confirmed in another study of 454 AML patients, this time comprising a cohort of cytogenetically abnormal (CA) patients [27]. Functional insight into the role of miR-181a in therapeutic resistance came from two studies that analyzed the resistance of AML cell lines to cytarabine and daunorubicin treatment, respectively [49,50]. Both studies demonstrated that resistant subclones were characterized by decreased miR-181a expression levels and that re-expression, as achieved by miR-181a mimics, was able to restore chemosensitivity. Interestingly, the *BCL2* genes were identified as direct miR-181a targets in both studies and the miR-181a induced chemosensitivity was shown to be mediated via this miR-181a/*BCL2* axis. Finally, an interesting therapeutic approach to overcome the therapeutic resistance induced by decreased miR-181a was presented by Hickey and coworkers [58]. They observed that high expression of miR-181a correlated with N-terminal mutations in *CEBPA*. These mutations account for approximately 90% of *CEBPA* mutations in AML and enable translation of a truncated *CEBPA*-p30 isoform. The authors could show that this isoform directly targets the miR-181a promoter and thereby causes its upregulation. Most importantly, however, lenalidomide, which is currently used for the treatment of multiple myeloma and myelodysplastic syndromes, induced the expression of *CEBPA*-p30 and miR-181a, thereby inhibiting leukemogenic effects in vitro and in vivo. The authors thereby provided a possible explanation for the favorable prognostic impact of mutated *CEBPA* on the one hand, and a possible therapeutic intervention to overcome therapeutic resistance via a miR-181a axis on the other hand.

Another interesting example constitutes the family of let-7 miRNAs. This miRNA family was first discovered in nematodes and describes one of the first human miRNAs described at all [59]. Downregulation of let-7f has been described in a doxorubicin-resistant subclone of K562 leukemia cells [28]. In a subsequent series of experiments, the authors performed artificial re-expression of this miRNA and measured doxorubicin IC_50_ values, defined as the doxorubicin concentration required to inhibit cell growth by 50%. Importantly, re-expression of let-7f in the doxorubicin-resistant subclone resulted in a significant decrease of IC_50_ values, thereby indicating a role of let-7f downregulation in development of anthracycline resistance. Indeed, when 60 patients with de novo and 50 with refractory AML were analyzed, let-7f expression levels turned out to be decreased in specimens from the refractory cohort. Chen and coworkers demonstrated that increased levels of let-7a cause enhanced sensitivity to cytarabine by downregulation of the BCL proteins and subsequent induction of apoptosis [51]. Importantly, however, the value of aberrant let-7a expression as prognostic marker still remains a matter of debate. Current data are conflicting in this respect [60,61], which might also be due to the fact that distinct let-7a family members have been reported [62]. The same problem seems to apply for other miRNAs as well, among others including miR-128, miR-331 and miR-27a, respectively [25,52,53,63]. All of these have been studied in functional in vitro and/or in vivo approaches and their overexpression has been shown to enhance sensitivity to cytotoxic agents used in high-intensity treatment approaches used in AML. However, association of these miRNA expression patterns with response rates in clinical datasets, which would further corroborate these functional observations, is either missing or contradictory. Larger, prospective and uniformly treated patient cohorts will definitely be needed to unambiguously clarify these issues and to delineate the role of these miRNAs in the development of therapeutic resistance. Whereas association with therapeutic resistance has been backed by functional in vitro and/or in vivo approaches in the miRNAs described so far, a group of potential candidate miRNAs exists, in which clinical data demonstrated that their increased expression levels are associated with a favorable prognosis but where functional data supporting these clinical correlations are still missing. Among others, these comprise miR-9* (miR-9-3p), miR-96, miR135a and miR-409 [29,30,31]. An interesting example from this group is the miR-10 family, which has been linked to mutations in *NPM1* previously [22,64]. In subsequent follow-up studies, miR-10 expression and *NPM1* mutations indeed correlated with higher CR rates in uni- and multivariate models, thereby making it a likely candidate for modulating therapeutic resistance [32]. However, when the authors employed functional analyses, including cytarabine treatment after miR-10 modulation, they did not see any impact of miR-10 overexpression. These data suggest that—although miR-10 and mutated *NPM1* seem to be linked—the favorable prognostic effects seem to be mediated primarily via *NPM1* and that miR-10 seems to be a bystander of these events. Furthermore, they clearly highlight the importance of functional experiments, in which the effects of miRNA modulation are tested after treatment with the respective cytotoxic agents. Relying solely on clinical correlations, i.e., the achievement of CR, might result in misinterpretation of the results.

While this part of the review focused on miRNAs categorized as members of group I, a large fraction has to be assigned to group II, which means that increased expression mediates therapeutic resistance, or, conversely, decreased expression relays sensitivity. One such example is miR-155. This miRNA has been shown to be upregulated in AML specimens carrying an internal tandem duplication within the *FLT3* gene (*FLT3*-ITD) [22,44,64,65]. Indeed, a causal relation could be shown, as the expression of miR-155 could be induced by the *FLT3*-ITD downstream targets nuclear factor kappa-light-chain-enhancer of activated B cells (NF-κB) and signal transducer and activator of transcription 5 STAT5. The role of miR-155 in the pathogenesis of AML has also been demonstrated in functional approaches. It has been shown to directly target the *PU.1* transcription factor, thereby promoting proliferation of myeloid cells on the one hand and inhibiting their apoptosis on the other hand [23]. miR-155 is also of interest from a therapeutic view, as its expression can be inhibited by Silvestrol, a compound isolated from the Indonesian plant *Aglaia foveolata* [66] and by the synthetic Nedd8-activating enzyme inhibitor MLN4924 [67]. Indeed, these approaches are able to induce anti-leukemogenic effects both in vitro and in vivo [66,67]. The prognostic value of miR-155, and particularly its association with therapeutic sensitivity, was first shown in a study comprising 363 leukemic specimens of newly diagnosed AML patients with a normal karyotype. Increased expression correlated with decreased CR rates and a shorter overall survival (OS) in both uni- and multivariate analyses within this cohort [34]. As all patients were treated with high-dose induction treatments, these data suggested a potential role of increased miR-155 expression in the development of therapeutic resistance. Again, high miR-155 expression correlated with *FLT3*-ITD and the expression of genes involved in NF-κB activation, which again highlights the existence of the *FLT3*-ITD/NF-κB/miR-155 axis described above. In a subsequent study comprising 138 AML patient specimens, high miR-155 expression was included in a prognostic scoring system, including information on the expression of specific miRNAs [35]. Again, this model proved to be of prognostic relevance as the subgroup with high miR-155 levels correlated with a shortened OS. The link to therapeutic resistance was further strengthened by data showing that a doxorubicin resistant subclone of K562 leukemia cells demonstrated significantly increased expression levels of miR-155 [68]. However, functional studies exploring the mechanisms of miR-155 induced chemoresistance in more detail are still missing. This is of particular relevance, as conflicting results in this area have been reported. In discordance with the results presented above, one study observed resistance to cytarabine in AML cells not by miR-155a overexpression but by employing its knockdown by the means of miRNA inhibitors [69]. Another miRNA, in which increased expression plays a role is miR-125b. This miRNA has been shown to exhibit increased expression in a variety of leukemias, including AML [70]. Furthermore, enforced expression of miR-125b has been shown to cause leukemia in murine transplant models [70]. Zhang and colleagues first demonstrated its involvement in therapeutic resistance, when studying 169 primary AML specimens (including 114 samples with acute promyelocytic leukemia [APL] as well as specimens obtained at diagnosis and following therapy) [36]. They observed that miR-125b expression was upregulated in diagnostic AML samples when compared to healthy controls. Importantly, however, while it was downregulated in post-therapeutic specimens in patients achieving a CR, it increased again in those showing relapsed disease. To gain more insight into this phenomenon, the authors performed cellular cytotoxicity assays in AML and APL cell lines with and without transfection of miR-125b and assessed IC_50_ values for treatment with doxorubicin and all *trans* retinoic acid (ATRA), respectively. Indeed, overexpression of miR-125b caused a significant increase in IC_50_ of both drugs, thereby confirming its role in development of therapeutic resistance. These data were subsequently corroborated in a cohort of 46 acute leukemia patients [37]. Again, high miR-125b expression was observed and associated with decreased event-free survival (EFS). In a series of AML cell lines, the authors demonstrated again that overexpression of miR-125b contributes to daunorubicin resistance, and that these effects are mediated via downregulation of *PUMA* and *GRK2*, which in turn resulted in decreased apoptosis. Another interesting example of increased miRNA expression conferring resistance is miR-126. This miRNA was originally identified as overexpressed in core binding factor (CBF) AML [71]. The authors could show that increased expression of miR-126 is associated with hypomethylation of its promoter region and causes leukemogenic effects in vitro. Leeuw and colleagues could subsequently show, that miR-126 expression was significantly increased in hematopoietic (HSC) and leukemic stem cells (LSC), when compared to more mature leukemic progenitors [38]. When analyzing 92 non-CBF AML patient specimens, high miR-126 expression proved to be an adverse prognostic marker, which was associated with poorer EFS, relapse-free survival (RFS) and OS. In functional experiments, miR-126 knockdown decreased the growth of AML cells by inducing apoptosis. Similar results were observed in a cohort of 126 AML patients treated with high-dose induction chemotherapy within the CALGB protocols [39]. High expression of miR-126 correlated with decreased CR rates, as well as with shortened EFS and OS in older patients >60 years. As previously demonstrated, the authors also showed that increased expression of miR-126 correlated with hypomethylation of its promoter as well, and that its expression was particularly increased within the LSC compartment. Most interestingly, the authors present a nanoparticle based miR-126 inhibitor, which enabled the pronounced downregulation of this miRNA, both in vitro and in vivo. Indeed, this approach caused strong anti-leukemic effects in murine leukemia transplant models. A real link to the development of resistance to chemotherapy was established by Shibayama and coworkers, who modified miR-126 expression in AML cell lines and subsequently assessed their sensitivity to cytarabine and idarubicin, respectively, in MTT assays [55]. While miR-126 overexpression had no effect on the sensitivity to idarubicin, it caused resistance to cytarabine, as evidenced by significantly increased IC_50_ values. More detail in the mechanisms behind these phenomena could be provided very recently by Lechman and colleagues [40]. In a landmark study, they could prove that, indeed, the LSC compartment exhibits the highest levels of miR-126 expression and could confirm the adverse prognostic impact of high miR-126 levels in AML patients. In a series of in vitro and in vivo approaches, they could demonstrate that miR-126 preserves LSC in a quiescent state by targeting the phosphoinositide 3-kinase/protein kinase B/mechanistic target of rapamycin (PI3K/AKT/MTOR) pathway, which in turn mediates resistance of LSC to daunorubicin. Despite all these progress, again caution has to be employed, when the value of miR-126 as therapeutic target is revisited. As already shown above for miR-155, a certain two-faceted role of miR-126 seems to exist, as a recent publication observed the same leukemogenic effects, both after overexpression and knockdown of miR-126 [72]. These results again highlight the fact that the regulation and effects of changes in miRNA expression are part of a complex network depending on a variety of co-factors, most of them hitherto unknown. Furthermore, they demonstrate the importance of thorough preclinical evaluation of miR-targeting drugs, before the step into clinical trials is dared. Beside these well studied miRNAs, again a group of candidates exists, in which functional relevance of their increased expression in the development of therapeutic resistance has been shown in vitro and/or in vivo, however, where clinical data corroborating these results are still missing or preliminary. Among others, these include miR-20a and miR-32 [33,54,56]. Finally, as already described above, a group of miRNAs in which increased expression could be linked to adverse outcome (or in which decreased expression was linked to favorable prognosis) in clinical association studies, but where functional experiments are missing, has been described. Among others, this group comprises miR-210, miR-3151, miR-196b, miR-644, miR-199a and miR-191 [31,41,42,43,44]. Further functional and or clinical association studies, respectively, will be needed for these two miRNA groups to unambiguously clarify their role in the development of therapeutic resistance.

### 2.2. The Role of lncRNAs

While a lot of data are available on the role of miRNAs in development of resistance to high intensity therapeutic regimens in AML, data about the role of lncRNAs within this field are scarce until now (Table 1). A first lead into this direction came from Garzon and colleagues in 2014, who studied the association of lncRNAs with prognosis in a cohort of 148 CN-AML patients treated within the CALGB trials by microarray expression profiling [73]. By selecting lncRNAs associated with EFS, the authors were able to establish a lncRNA-based scoring system, which included the expression status of 48 lncRNAs and which was able to accurately predict CR, disease-free survival (DFS) and OS rates. The reliability of this score could subsequently be validated in a second cohort of 71 AML patients, where lncRNA expression was studied by an independent technique (RNA sequencing). Again, the lncRNA score correlated with CR, DFS and OS in uni- and multivariate analyses. Although no functional analyses were included, the fact that lncRNA expression profiles correlated with CR rates in patients treated with similar high-dose approaches suggested a potential involvement of lncRNAs in the development of therapeutic resistance for the first time.

A lncRNA of potential interest might be Hox transcript antisense intergenic RNA (HOTAIR), which displays a length of 2.2 kb and which is expressed from the *HOXC* locus on chromosome 12 [74,75]. Aberrant expression of HOTAIR has been shown in a variety of solid tumors and its involvement in malignant transformation could be proven in functional in vitro and in vivo approaches [74]. Recently, increased expression of HOTAIR was observed in AML as well. Xing and colleagues studied the expression of HOTAIR in 136 diagnostic AML specimens and compared the results to normal controls. They observed a significant upregulation of HOTAIR in AML and could prove the functional involvement of HOTAIR in AML cell growth, apoptosis and colony formation by means of knockdown experiments. Finally, when looking on clinical parameters, high expression of HOTAIR correlated with worse DFS and OS [45]. Similar results were observed in two additional studies [46,47]. Again, high expression of HOTAIR was associated with an adverse clinical course and proved to be involved in malignant transformation of hematopoietic cells. Although a definitive link to resistance to therapeutic high-intensity AML treatment regimens is still missing, HOTAIR induced resistance to anthracyclines in functional in vitro models of solid tumors previously [76]. It will be of interest to see whether these mechanisms apply to AML as well. Another lncRNA showing at least a clinical indication to therapeutic resistance is Hox antisense intergenic RNA myeloid 1 (HOTAIRM1). In an analysis of 241 AML patient specimens, increased levels of HOTAIRM1 were observed and correlated with shorter OS, shorter leukemia-free survival and a higher cumulative incidence of relapse in uni- and multivariate analyses [48]. Again, functional experiments further proving that therapeutic resistance is indeed mediated via HOTAIRM1 will be needed to further clarify the role of HOTAIRM1 in this respect.

## 3. miRNAs and lncRNAs in Sensitivity and Resistance to Low-Intensity Therapeutic Regimens

Although a subset of patients can be cured with high-dose chemotherapy and/or allogeneic hematopoietic stem cell transplantation, a large subset of AML patients are ineligible to these approaches, mainly because of older age and/or comorbidities [1,2,6]. While best supportive care was the only treatment option in these cases for a long time, the introduction of LITR therapies was able to significantly increase both OS and quality of life in affected patients [1,9,77]. The first LITR to be introduced was low-dose cytarabine (LDAC). At a dose of only 20 mg twice daily for 10 days every four weeks, it demonstrated increased survival as compared to best supportive care only [78]. While LDAC served as the mainstay of AML LITR for a long time, the recent introduction of HMA treatments remarked a further significant development in this area [1,8], with the cytidine analogues azacitidine (5-azacytidine) and decitabine (5-aza-2′-deoxycytidine) representing the currently best studied substances within this category. Two main anti-tumorigenic effects of these drugs have been described [8]: (a) Both drugs act as antimetabolites and are incorporated into DNA (decitabine) or DNA and RNA (azacitidine), respectively. Consequently, DNA damage response is induced; (b) Both drugs cause hypomethylation of DNA by inhibiting DNA methyltransferases, which is thought to restore the expression of silenced tumor suppressor genes. Both drugs have been tested in multicenter, randomized phase III trials, where they have shown efficacy when compared to LDAC, BSC and/or intensive chemotherapy [8]. Consequently, these drugs evolved as mainstay of AML treatment in older patients who are ineligible to high-intensity chemotherapy, thereby causing a significant amelioration of survival within this subgroup. However, despite all this progress in the development of LITR approaches, a significant proportion of patients exhibits refractory disease. Furthermore, even in patients showing partial or complete remission of AML under LITR therapy, development of therapeutic resistance is observed in the vast majority of patients, thereby causing recurrence or progression of AML [1,8]. Hence, research on strategies overcoming the mechanisms behind this therapeutic resistance is desperately needed to further improve survival within these patients.

### 3.1. The Role of miRNAs

**Figure 2:**
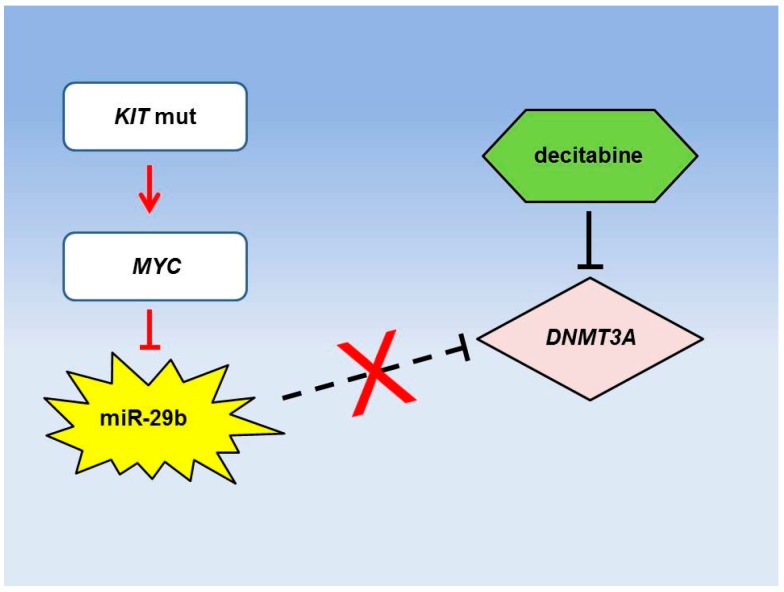
Example of a miRNA network influencing therapeutic sensitivity in AML low-intensity treatment. Gain of function mutations of *KIT* occur in AML and have been shown to decrease miR-29b expression levels via upregulation of the *MYC* oncogene. As a result, downregulation of the miR-29b target *DNMT3A* is prevented. This is of relevance for decitabine treatment, which has been shown to inhibit *DNMT3A* activity and which has proven to be more efficient, when *DNMT3A* levels are low [79,82,84].

Aberrant expression of miRNAs has been associated with differential sensitivity to HMA treatment in AML (Table 2). The probably best studied example is miR-29b (Figure 2). This miRNA has been shown to be downregulated in AML via activation of a *KIT-MYC* axis [79] and has been demonstrated to be involved in the regulation of DNA methylation by directly targeting DNA methyltransferases 3A (*DNMT3A*) and 3B (*DNMT3B*) [80]. Indeed, artificial overexpression of miR-29b caused inhibition of proliferation of AML cells in vitro and of leukemogenesis in vivo [81]. In a phase II clinical trial of decitabine in older AML patients, Blum and coworkers studied miR-29b expression in diagnostic specimens and observed that increased levels of miR-29b correlated with superior clinical response to decitabine treatment [82]. Likewise, increased expression of miR-29b did also correlate with decreased expression levels of *DNMT3A*. To further study this phenomenon, the same group performed overexpression of miR-29b using a transferrin-conjugated nanoparticle delivery system for synthetic miR-29b in AML cell lines and primary patient specimens followed by treatment with decitabine [83]. Interestingly, overexpression of miR-29b increased the sensitivity to decitabine thereby increasing the anti-leukemic properties of this drug in in vitro and in vivo assays. They further observed that miR-29b expression in AML is repressed via histone deacetylases (HDACs) and can be restored by the application of HDAC inhibitors [84]. Indeed, sequential treatment with HDAC inhibitors and decitabine resulted in stronger anti-leukemic activities in vitro and in vivo as compared to either drug alone.

Another member of the miR-29 family studied in this respect is miR-29c. Butrym and colleagues studied miR-29c in AML patients treated with azacitidine and observed increased expression of this miRNA in AML specimens when compared to a healthy control group [85]. Unexpectedly, lower expression of miR-29c correlated with good clinical response to azacitidine, which is in sharp contrast to the findings observed in miR-29b. It will be of interest to delineate whether these discrepancies are due to differences between azacitidine and decitabine, or whether they are due to differences between these miR-29 subforms. Beside the miR-29 family, miR-331 could be associated with chemosensitivity in LITR treatment of AML. Butrym and colleagues studied its expression in 95 bone marrow specimens of newly diagnosed AML patients, who were treated either with intensive (*n* = 56), or non-intensive therapies (*n* = 27) or best supportive care (*n* = 12) [63]. Among the non-intensively treated patients, LDAC and azacitidine were used. Interestingly, increased expression of miR-331 correlated to lower CR rates and a greater risk of death. Unfortunately, functional data proving that miR-331 indeed plays a causal role in chemoresistance to azacitidine are still missing, therefore, these experiments will be important for future research within this area.

Another interesting group of miRNAs for HMA treatment encompasses miRNAs that are suppressed in AML by hypermethylation occurring within their promoter regions. Many of these miRNAs fulfill a tumor-suppressor function under normal circumstances, as they directly target and thus inhibit a broad range of oncogenes. Consequently, their suppression in AML results in increased oncogenic activity, thereby aggravating the leukemogenic potential. In light of the fact that treatment with the hypomethylating agents azacitidine and decitabine might restore the expression of these miRNAs, analysis of their pre-treatment expression levels might provide important information on patients profiting most from HMA therapy. One such example is miR-193a, which has been shown to be silenced in AML by methylation [86]. Additionally, it has been demonstrated to target the *KIT* oncogene. Treatment of AML cells characterized by activating *KIT* mutations and/or *KIT* overexpression with azacitidine caused the re-expression of this miRNA, which in turn downregulated the *KIT* oncogene and inhibited cellular growth [86]. miR-663 has also been shown to be hypermethylated in both AML and chronic myeloid leukemia (CML) [87,88]. Again, this induced oncogenes, such as *HRAS*, and leukemogenic transformation [87]. Importantly, treatment with hypomethylating agents was able to reverse this miR-663 silencing [88]. Other examples are miR-125a and miR-370. Both have been shown to be methylated and suppressed in AML, which in turn resulted in activation of the ErbB pathway in the case of miR-125a [89] and the transcription factor *FoxM1* in the case of miR-370 [90]. Importantly, however, treatment with hypomethylating agents was able to reverse these effects. miR-124-1 is a tumor suppressor miRNA downregulated in various hematologic malignancies including AML [91]. Again, treatment with decitabine was able to restore its expression and caused the downregulation of miR-124-1 target genes, including *CDK6* [91]. Evidently, clinical studies demonstrating that AML patients with decreased expression of these miRNAs truly exhibit a particular benefit from HMA treatment are still missing. Answering these questions within prospective clinical trials will be of interest and is currently addressed, as miRNA expression profiling is increasingly introduced into clinical trials, also including those with LITR treatment.

Another possibility of chemoresistance mediated by hypermethylation of miRNAs has recently been observed in CML. miR-217, miR-23a and miR-203 are miRNAs that frequently show a hypermethylated promoter region and decreased expression within this malignancy [94,95,96]. Importantly, this mechanism has been shown to confer resistance to tyrosine kinase inhibitors, the current gold-standard in CML treatment [95]. Simultaneous treatment with hypomethylating agents restored the expression of these miRNAs [92,95], which in turn re-sensitized the cells to TKI treatment [95]. Consequently, the hypothesis that resistance to standard induction therapy of AML might be caused by the epigenetic inactivation of miRNAs as well and therefore might be overcome by simultaneous administration of HMA is appealing. These hopes were further nurtured by both in vitro and in vivo data, where synergistic effects between cytarabine and azacitidine could be shown, and particularly, where cytarabine-resistant cell lines could be re-sensitized by simultaneous administration of azacitidine [97,98]. Additionally, data from a clinical phase 1 study analyzing a combination of decitabine with standard induction chemotherapy suggested its use to be safe and feasible [99]. Unfortunately, however, the authors of a large and randomized phase II trial, who studied the combination of azacitidine and standard induction chemotherapy in older AML patients, failed to observe a beneficial effect of this regimen [100]. It will be interesting to see, whether these results can be ameliorated by altered dosing schedules or by the use of newer hypomethylating agents, such as guadecitabine [101]. Another obstacle in interpretation of the CML data mentioned above, is that AML constitutes a distinct genetic disease. Whereas miR-203 shows downregulation in approximately 10% of AML patient samples as well [92], this does not apply for miR-23a. In previous work of our own and other groups, including both in vitro and in vivo approaches, miR-23a was demonstrated to act rather as oncogene within this disease [21,102]. From a mechanistic point of view, increased expression of miR-23a caused a downregulation of the tumor- and metastasis suppressor *RKIP* thereby increasing the proliferation of leukemic cells. Taken together, these results clearly demonstrate that different hematologic neoplasms are characterized by distinct clonal architectures and that, although the concept of hypermethylated miRNAs conferring resistance to therapy might apply for all of them, the potential culprit miRNAs have to be identified in unbiased approaches specifically addressing each malignancy separately.

### 3.2. The Role of lncRNAs

Much less is known about the role of long non-coding RNAs within the process of sensitivity to LITR, in particular HMA treatment (Table 2). Treppendahl and colleagues studied the 106-nucleotide lncRNA vault RNA2-1 (vtRNA2-1; previously mislabeled as miR-886) in AML [93]. They observed its frequent methylation in AML patient specimens, which correlated to decreased expression of this lncRNA and worse clinical outcome. Importantly, treatment with azacitidine was able to restore vtRNA2-1 expression and to down-regulate the phosphorylated RNA-dependent protein kinase (pPKR), a known vtRNA2-1 target that has been shown to promote cell survival in AML. As for miRNAs, prospective clinical trials specifically addressing this question will be needed to truly prove that patients with methylation of vtRNA2-1 demonstrate a special profit from HMA treatment.

## 4. Conclusions and Outlook

ncRNAs are increasingly recognized as central players in development of therapeutic resistance to cytotoxic agents administered for the treatment of human malignancies. In this review, we discuss miRNAs and lncRNAs involved in therapeutic resistance and sensitivity to intensive and non-intensive treatment regimens currently used in AML. Firstly, this is of relevance as prognostic risk stratification in AML has improved dramatically within recent years by inclusion of cytogenetic and molecular markers [1,2,7,103]. This has led to treatment algorithms, where the molecular profile of individual patients is able to predict their response to treatment, and consequently, where intensity and duration of therapy is routinely planned according to this genetic information [1]. It seems a likely option that these risk stratification schemes could be further improved by the inclusion of selected ncRNA expression profiles. Additionally, however, it is a tempting vision, that therapeutic sensitivity to conventional chemotherapy can be restored or further increased by the artificial modulation of ncRNA expression(s) in potential future therapeutic approaches. The likeliness of such a scenario is further supported by the fact that therapeutic targeting of miRNAs has become a feasible option and that the first miRNA therapeutics are already available [104]. Although this sounds promising, it has to be mentioned that beside the specific disease-modifying effects, these substances seem to come along with unique side effect profiles as well. These can also be severe, as in the case of the miR-34 mimic MRX34, where a phase 1 trial was recently halted following multiple immune-related severe adverse events (http://investor.mirnarx.com/releases.cfm; 20 September 2016). Therefore, although ncRNA therapeutics are promising new drugs with the potential to overcome therapeutic resistance in AML, their establishment as therapeutic agents that can be safely used in the clinical routine setting, is still at its beginning. To achieve this goal, ongoing research will be needed in identification of the most promising ncRNA targets on the one hand and design of the best ncRNA therapeutics with appropriate efficacy and safety profiles on the other hand. Finally, it will also be important to identify the ideal cohort of patients that truly benefit from a specific ncRNA-targeting therapeutic agent.

## Figures and Tables

**Table 1:** Non-coding RNAs (ncRNAs) linked to therapeutic resistance and sensitivity, respectively, to high intensity treatment regimens in acute myeloid leukemia (AML).

ncRNA Groups and Data Categories	ncRNA	Reference
Group I—clinical data: high expression associated with sensitivity	miR-181a	[25,26,27]
	let-7f	[28]
	miR-9* (miR-9-3p)	[29]
	miR-96	[30]
	miR-135a	[31]
	miR-409	[31]
	miR-10	[32]
	(miR-20a)	[33]
Group II—clinical data: high expression associated with resistance	miR-155	[34,35]
	miR-125b	[36,37]
	miR-126	[38,39,40]
	miR-210	[41]
	miR-3151	[42,43]
	miR-196b	[31]
	miR-199a	[44]
	miR-191	[44]
	miR-644	[31]
	(miR-128)	[25]
	HOTAIR	[45,46,47]
	HOTAIRM1	[48]
Group I—experimental data: high expression inducing sensitivity	miR-181a	[49,50]
	let-7f	[28]
	let-7a	[51]
	miR-128	[52]
	miR-331	[53]
	miR-27a	[53]
Group II—experimental data: high expression inducing resistance	miR-125b	[36,37]
	miR-32	[54]
	miR-126	[40,55]
	miR-20a	[56]

**Table 2:** ncRNAs linked to therapeutic resistance and sensitivity, respectively, to low intensity treatment regimens of AML.

ncRNA	Characteristics	Reference
miR-29b ^1^	Increased expression associated with favorable clinical course	[82]
	Overexpression increases sensitivity to decitabine in functional assays	[83]
miR-29c ^2^	Decreased expression associated with favorable clinical course	[85]
miR-331 ^2,3^	Increased expression associated with unfavorable clinical course	[63]
miR-193a ^2^	Silenced by promoter methylation; reversed by HMA treatment	[86]
miR-663 ^2^		[87,88]
miR-125a ^1^		[89]
miR-370 ^1^		[90]
miR-124-1 ^1^		[91]
miR-203 ^1^		[92]
vtRNA2-1 ^1,2^		[93]

Low intensity treatment regimens evaluated: ^1^ decitabine; ^2^ azacitidine; ^3^ LDAC.
